# Is Bleaching Effective in Managing Post-orthodontic White-spot Lesions? A Systematic Review

**DOI:** 10.3290/j.ohpd.a44113

**Published:** 2020-04-01

**Authors:** Sotiria Gizani, Dimitrios Kloukos, Aikaterini Papadimitriou, Theoni Roumani, Svante Twetman

**Affiliations:** a Professor, Department of Paediatric Dentistry and Orthodontics, Dental School, National and Kapodistrian University of Athens, Greece. Wrote and approved the manuscript.; b Senior Lecturer, Department of Orthodontics and Dentofacial Orthopaedics, School of Dental Medicine, University of Bern, Switzerland; Head, Department of Orthodontics and Dentofacial Orthopaedics, 251 Hellenic Air Force and VA General Hospital, Athens, Greece. Literature search, all methodological procedures, read and approved the manuscript.; c Postgraduate Student, Department of Orthodontics, Faculty of Dentistry, School of Health Sciences, Aristotle University of Thessaloniki, Thessaloniki, Greece. Data extraction, quality assessement of included studies, read and approved the manuscript.; d Paediatric Dentist in Private Practice, Nea Smirni, Greece. Data extraction, quality assessement of included studies, read and approved the manuscript.; e Professor, Department of Dentistry, Faculty of Health and Medical Sciences, University of Copenhagen, Denmark. Project supervisor, read and approved the manuscript.

**Keywords:** bleaching, enamel demineralisation, fluoride, tooth whitening, white spot lesions

## Abstract

**Purpose:**

White spot lesions (WSL) are common side-effects of orthodontic treatment with fixed multi-bracketed appliances. The aim of this review was to find all available literature and critically assess the evidence for the efficacy of bleaching as a method to treat or alleviate post-orthodontic WSLs in permanent teeth.

**Materials and Methods:**

Electronic databases were screened for relevant literature with the aid of predetermined search strategies. All types of studies, including randomised or nonrandomised controlled trials (RCTs or CCTs), prospective and retrospective studies, as well as in vitro studies were considered eligible for inclusion. The reference lists of all included articles were hand searched for additional studies. Two authors independently performed study selection, data extraction, and risk of bias assessment.

**Results:**

One RCT and 8 in vitro studies met the inclusion criteria. Seven studies were classified as having a high risk of bias while 2 in vitro studies were graded as having a moderate risk of bias. The results showed that bleaching of WSL can diminish colour disparities between carious and non-affected areas, but the certainty of the evidence was very low. The high degree of methodological heterogeneity precluded a valid interpretation of the results through pooled estimates.

**Conclusions:**

The findings from the present systematic review could not support or refute bleaching as an effective method for management of post-orthodontic WSLs. Because most of the studies in this field are in vitro and solid scientific evidence of low risk of bias is scare, further prospective in vivo studies are necessary.

Although fixed appliances have revolutionised contemporary orthodontic treatment, they are at the same time a risk factor for the integrity of tooth enamel. This is mainly attributed to plaque accumulation and the subsequent development of bacterial colonies.^44^ The placement of fixed orthodontic appliances interferes with standard oral hygiene procedures and causes alterations in the oral microflora by reducing pH and increasing bacterial affinity to the metallic surfaces due to electrostatic reactions.^1^ White spot lesions (WSL) can develop as soon as 4 weeks after placement of fixed orthodontic appliances,^30^ with a prevalence ranging from 5% to 97% at the time of debonding.^7,17^ The clinical appearance is an opaque white area of demineralised enamel that follows the shape of the bracket.^32^ Although WSL have the ability to remineralise after bracket removal,^39^ with the greatest remineralisation within the first months,^21^ they may in some cases still be detected up to 5 years after debonding.^34^

The management of WSL has traditionally focused on applying remineralising agents such as topical fluoride, amorphous calcium phosphate and self-assembling peptides.^41^ However, some researchers^33^ have warned against the use of highly concentrated fluorides, since rapid surface hypermineralisation may block deeper remineralisation of the subsurface lesion. Clinically, this may lead to a persistent intensive whitish/opaque appearance of the lesion. It is therefore suggested that slow, natural remineralisation through saliva and low-fluoride exposure may produce a more aesthetically pleasing result.^26^

Tooth bleaching has been suggested as an option for managing WSL. This intervention has primarily been applied to teeth with fluorosis, and a better colour match has been reported after treatment of maxillary incisors with a 35% hydrogen peroxide gel.^9^ Bleaching is a complex oxidation process, in which reactive forms of oxygen penetrate through the pores of enamel rods to reach the dentin.^43^ Reported side-effects include decreased wear resistance of enamel and dentin, increased surface roughness and decreased microhardness, as well as histomorphologic changes.^6,14^ Most of the literature is, however, based on in vitro studies using artificial lesions, which impedes extrapolation of data to the clinical setting. In a recent systematic review,^40^ no clinical trials concerning bleaching of post-orthodontic WSL fulfilled the inclusion criteria. Taking the growing aesthetic demands of orthodontic patients into account, as well as the need for minimally invasive approaches,^37^ a systematic review on bleaching including laboratory data was therefore indicated.

The aim of the present systematic review was to identify and critically assess the efficacy of bleaching as a method to treat or alleviate post-orthodontic WSL in permanent teeth. The primary research question was: ‘Is bleaching, alone or in combination with other methods effective in managing post-orthodontic white spots and initial caries lesions?’ The secondary question was ‘Is tooth bleaching associated with side-effects or increased risk of enamel demineralisation?’

## Materials and Methods

This systematic review was conducted in accordance with The Cochrane Handbook for Systematic Reviews of Interventions.^19^ PICO was set as follows:

- Population: patients with permanent teeth, or extracted permanent human teeth, with natural or artificial initial white spot caries lesions.
- Intervention: bleaching or whitening with any chemical agent, alone or in combination with any other technique or agent such as laser treatment, fluoride applications, resin infiltration or micro-abrasion.
- Control: placebo or no treatment.
- Outcome: visual appearance or area of lesions after clinical scoring or fluorescence, enamel hardness and/or microradiography.

Any study design was considered eligible for inclusion in this review, including randomised controlled trials (RCTs), nonrandomised or quasi-randomised controlled trials, prospective and retrospective studies, as well as in vitro studies. Only studies with > 10 patients or teeth were considered eligible. Animal studies, case series and case reports were excluded. Studies with fluorotic or bovine teeth were excluded as well as interventions on interproximal, occlusal, and pit-and-fissure lesions.

### Search Methods for Identification of Studies

For identification of relevant literature, the following electronic databases were searched through July 1st, 2018, without date restrictions: MEDLINE (via Ovid and PubMed; Appendix), EMBASE (via Ovid), the Cochrane Oral Health Group’s Trials Register and CENTRAL. Detailed search strategies were developed for each database, based on MEDLINE, but were revised for each database to take differences in controlled vocabulary and syntax rules into account. Unpublished literature was searched on ClinicalTrials.gov, the National Research Register, and Pro-Quest Dissertation Abstracts and Thesis database. There were no language restrictions. The reference lists of all eligible studies were hand searched for additional studies.

### Selection of Studies

Two authors assessed the titles and abstracts of potentially eligible studies independently. When in doubt, full-text papers were ordered and evaluated by two authors. Disagreements were solved through discussion with the third author.

### Data Extraction and Management

Two review authors extracted data independently and any disagreements were resolved by consulting the third review author. The following data were tabulated: author, year, and title of the study, study design, setting, inclusion criteria, type of white spot lesion (natural/artificial), participants in intervention and control groups (number, age, gender), follow-up period, main outcome assessment (method/tools), secondary outcome assessment (method/tools), results and conclusions according to the authors. If stated, sources of funding, trial registration, and publishing of the trial’s protocol were recorded in order to make a more thorough assessment of heterogeneity and the external validity of the included trials. The preferred reporting items for systematic reviews and meta-analyses^31^ (PRISMA) were followed. The protocol of this study was not registered in a publicly accessible database.

### Assessment of Risk of Bias in Included Studies

Risk of bias in individual studies was assessed in accordance with the Cochrane Risk of Bias tool.^19^ For the in vitro reports, the tool was modified to the following domains: comparability of experimental conditions (selection bias), blinding of assessors (performance bias), losses or non-inclusion of specimens (attrition bias), selective reporting (reporting bias), and other bias.

An overall assessment of the risk of bias (high, moderate or low) was made for each included study; a) studies with at least one item designated to be at high risk of bias were regarded as having an overall high risk of bias; b) reports with an unclear risk of bias for one or more key domains were considered to be at moderate risk of bias; c) studies with a low risk of bias in all domains were rated as low risk of bias.

### Measures of Treatment Effect

All time points during the follow-up period were recorded. Decisions on which time-of-outcome assessment to use from each study were based on the most commonly reported time point among the included studies.

### Unit of Analysis and Missing Data

We anticipated that some of the included studies would present data from repeated observations on participants, which could lead to unit-of-analysis errors. In this event, the advice provided in the Cochrane Handbook for Systematic Reviews of Interventions^19^ was applied.

In studies where data were unclear or missing, we contacted the principal investigators or the corresponding author, or both.

### Assessment of Heterogeneity and Reporting Bias

We assessed clinical and methodological heterogeneity by examining the characteristics of the studies, the similarity between the types of participants, and the interventions and outcomes as specified in inclusion criteria for considering studies for this review.

Reporting biases arise when the reporting of research findings is affected by the nature or direction of the findings themselves. In the event that more than 10 studies with a comparable outcome are included, funnel plots are constructed and analysed for asymmetry.^13^

### Data Synthesis

We planned to conduct a meta-analysis if there were comparable studies reporting similar outcomes. If there were, risk ratios would have been combined for dichotomous data using fixed-effect models, unless there were more than 3 studies in the meta-analysis, when random-effects models would have been used.

## Results

### Description of Studies

**Fig 1:**
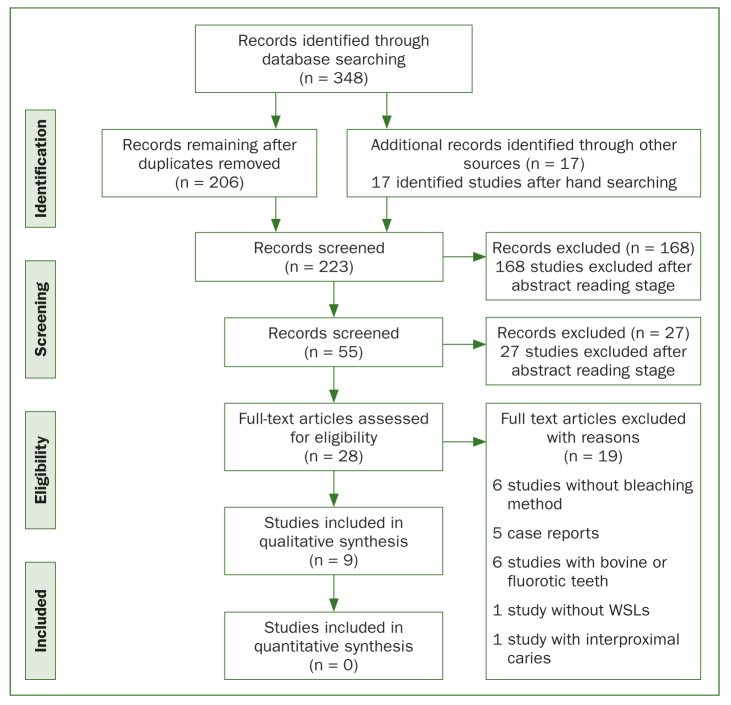
Study flow diagram.

In total, 348 studies were retrieved from the electronic searches. After excluding all duplicates, abstracts, and full texts not meeting the inclusion criteria, 28 studies were found. Of these, 19 were excluded after full-text reading. Finally, 9 studies were considered eligible for inclusion in this review: one RCT^24^ and 8 laboratory studies.^2,3,8,10,16, 23,35,38^ A flow-chart of this process is presented in Fig 1. The data extracted from the included studies are shown in Tables 1a and 1b.

**Table 1a:** Data extracted from included studies and study characteristics

First author, year	Study design	Country	Primary aim	Inclusion criteria	Type of lesion	Intervention groups (HP: hydrogen peroxide; CP: carbamide peroxide)	Participants (teeth) in intervention group	Control group	Participants (teeth) in control group
Ashnagar, 2017	In vitro	Iran	To evaluate whether or not conventional or laser activated bleaching predispose to caries	Erupted human third molars stored in thymol solution	Artificial, pH cycling	Conventional bleaching with 40% HP for 20 minutes each time. Diode laser-assisted bleaching. Nd:YAG laser-assisted bleaching. Bleaching was followed by pH cycling	15 molars per group	No treatment	15 molars
Kim, 2016	In vitro	South Korea	To evaluate the effect of bleaching on teeth with white spot lesions	Human maxillary premolars, sectioned into four parts	Artificial, pH cycling	Bleaching with 10% CP. Caries formation + bleaching. Caries formation + bleaching + remineralisation. Caries formation + remineralisation.	10 enamel sections in group 1; 20 enamel sections in groups 2-4	Only caries formation	10 enamel sections
Bollineni, 2014	In vitro	India	To evaluate the effect of adding fluoride to self-applied carbamide peroxide whitening gel on remineralisation of initial caries-like lesions	Freshly extracted third molars without defects, stored in PBS buffer	Artificial, acidified gel	Group A: demineralised + 10% CP bleaching (n=24). Group B: demineralised + 0.463% NaF + 10% CP bleaching (n=24). Group C: demineralisation only (n=24).	24 molars, sectioned in quadrants, one quadrant per group	Group D: no treatment	N = 24 molar quadrants
Cavalli, 2011	In vitro	Brazil	To evaluate the effect of adding fluoride and calcium to home-bleaching products on enamel mineral loss	Sound, extracted human third molars, stored in deionised water with thymol	Artificial	Commercial 10% CP gel, pH 6.8. Experimental 10% CP gel with 0.2% F, pH 6.8. Experimental 10% CP gel with 0.2% Ca, pH 6.5. Commercial 10% CP gel with 0.11% fluoride, pH 7.3. Commercial 10% CP gel with 0.11% fluoride, pH 6.4.	10 enamel slabs per group	Placebo gel	10 enamel slabs
Pinto, 2009	In vitro	Brazil	To study the effect of bleaching on hardness and morphology of sound enamel and on enamel with early caries lesions	Erupted human third molars, stored in saturated thymol solution	Artificial lesions, pH-cycling (group 3-6), stored in artificial saliva (group 1,3,4)	Sound enamel bleached with CP. Sound enamel, bleached with CP and pH cycled. Carious enamel bleached with CP. Carious enamel and pH cycled. Carious enamel treated with placebo gel and pH cycled. Carious enamel, bleached with CP and pH cycled.	10 enamel blocks per group	Group 5	10 enamel blocks
Knösel, 2007	RCT	Germany	To evaluate the effect of external bleaching on the colour of post-orthodontic white spot lesions	Maxillary incisors and canines	Inactive WSL	Office bleaching 60 min, 14 days break, home bleaching 1 h/day for 14 days.	10 participants	No bleaching	9 participants
Alves, 2007	In vitro	Brazil	To assess the influence of bleaching on the susceptibility of developing caries-like lesions	Human third molars	Artificial, pH cycling after bleaching	G1: home bleaching with 10% CP for 4 weeks. G2: home bleaching with 16% CP for 4 weeks. G3: in-office bleaching with 37% CP activated by a halogen light curing unit. In-office bleaching with 35% HP activated by light-emitting diode and laser energy.	30	G5: pH cycling, without bleaching. G6: no bleaching, no bleaching pH cycling.	10
Gladwell, 2006	In vitro	USA	To evaluate whether a whitening system with fluoride could remineralise previously demineralised enamel	Extracted third molars, sectioned into quadrants	Artificial, with acid gel	Group A: demineralised + 10% CP gel. Group B: demineralised + 10% CP gel with fluoride. Group D: demineralised, no bleaching.	24 enamel blocks per group	Group C: untreated, not-demineralised	24 enamel blocks
Pretty, 2005	In vitro	England	To evaluate if bleached enamel had an increased risk of either acid erosion or early caries	Human incisors	Artificial, demineralising solution	10% CP gel. 16% CP gel. 22% CP gel. 10% CP gel with xylitol, fluoride and potassium.	6 teeth per group	Half of each subsample was not bleached	6

**Table 1b:** Data extracted from included studies and study results

First author, year	Main outcome assessment	Follow-up	Drop-outs/attrition	Main results	Author’s main conclusions
Ashnagar, 2017	1. Knoop microhardness 2. DIAGNOdent (DD)	Not stated	None	All groups had a significant reduction in microhardness values but no significant differences between the groups. DD values were significantly reduced in the conventional and diode laser groups.	Bleaching with conventional or laser-activated technologies does not make teeth vulnerable to caries development.
Kim, 2016	1. Colour (spectro-radiometer) 2. Mineral content (EPMA) 3. Knoop microhardness	Baseline and after 14 days of bleaching	None	Bleaching of carious enamel extended whiteness without additional mineral loss. Treatment with CPP-ACP paste increased calcium, phosphate, and fluoride content in the lesion area and correlated well with microhardness.	Bleaching reduced colour disparities between sound and carious enamel without deteriorating the chemical and mechanical properties. The remineralising agent enhanced deposition of mineral in subsurface lesions.
Bollineni, 2014	1. Colour (Vita 3D Master Shade guide) 2. Lesion depth (polarised light microscopy)	Up to 21 days after bleaching	None	Significantly deeper lesions in groups A and C compared with Group B. Whitening data not presented.	Fluoride added to bleaching gel showed a remineralising effect on demineralised enamel (white spot lesions).
Cavalli, 2011	1. Mineral content (FT-Raman spectroscopy) 2. Knoop microhardness 3. Lesion depth (Polarised light microscopy)	After bleaching and after 14 days	None	Carbamide peroxide treatment decreased mineral content (subsurface mineral loss) and increased lesion depth. Inorganic deficit could be controlled by adding fluoride and calcium to the bleaching agent.	Addition of F and Ca to home-applied bleaching agents may reduce enamel mineral loss.
Pinto, 2009	1. Knoop surface microhardness 2. Morphology (SEM)	Not stated	None	Baseline mean microhardness values were similar in all groups. Groups exposed to enamel demineralisation (3-6) did not differ from each other and bleaching treatment reduced microhardness compared to the baseline. Changes in enamel morphology after treatments were observed for all groups.	CP bleaching promoted mineral loss of sound enamel but did not exacerbate mineral loss of the carious enamel.
Knösel, 2007	1. Colour determination (colourimeter) 2. Patient satisfaction (questionnaire)	28 days	None	Lightness of both sound enamel and WSLs was significantly higher after treatment. All patients in the bleaching group were satisfied with the outcome.	External bleaching can satisfactorily camouflage post-orthodontic WSLs.
Alves, 2007	1. Visual scoring of lesions (score 0-3) by three independent examiners	Not stated	None	G1 and G2: median score 1. G3, G3 and G5: median score 2. G6: median score 0.	Home bleaching reduced susceptibility to caries. In-office bleaching did not influence the development of caries.
Gladwell, 2006	1. Colour shade (Vita shade guide) 2. Histology (light microscopy)	After 21 treatments	None	Bleaching gels with and without fluoride had similar whitening effect. Differences in lesion depths were significantly reduced in group B.	Addition of fluoride to commercially available whitening gels enhance remineralisation without altering the whitening properties.
Pretty, 2005	1. Colour (Vita shade guide, Shade-Eye colourimeter) 2. QLF (erosion) 3. Transverse micro-radiography	Not stated	None	No significant differences in whitening effects between the gels. No significant differences in mineral loss between the groups. Demineralisation increased with time with a linear relationship to bleaching time of CP exposure.	Bleaching with carbamide peroxide gel did not increase the susceptibility of enamel to acid erosion or caries. The addition of xylitol, fluoride and potassium did not have an adverse effect on bleaching efficacy when compared to standard CP gels.

### Quality Assessment

**Fig 2:**
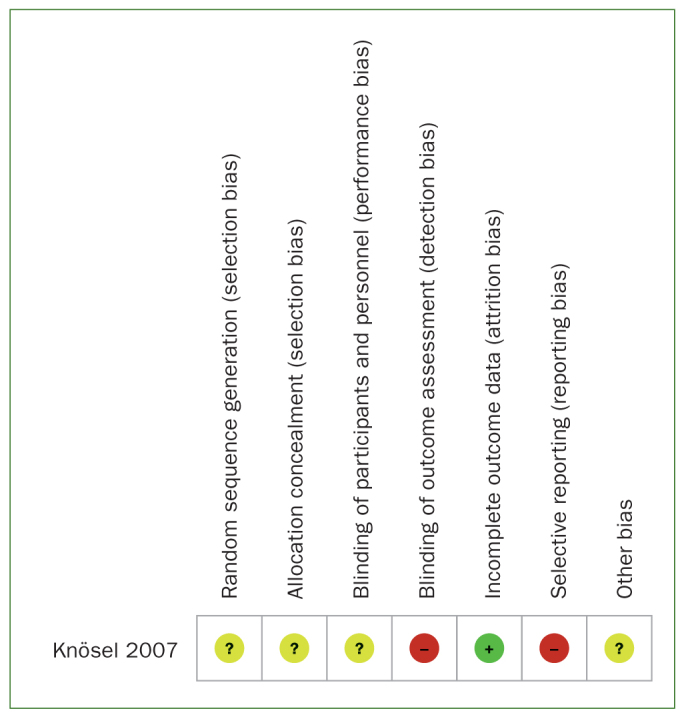
Risk of bias summary for the in vivo study.

**Fig 3:**
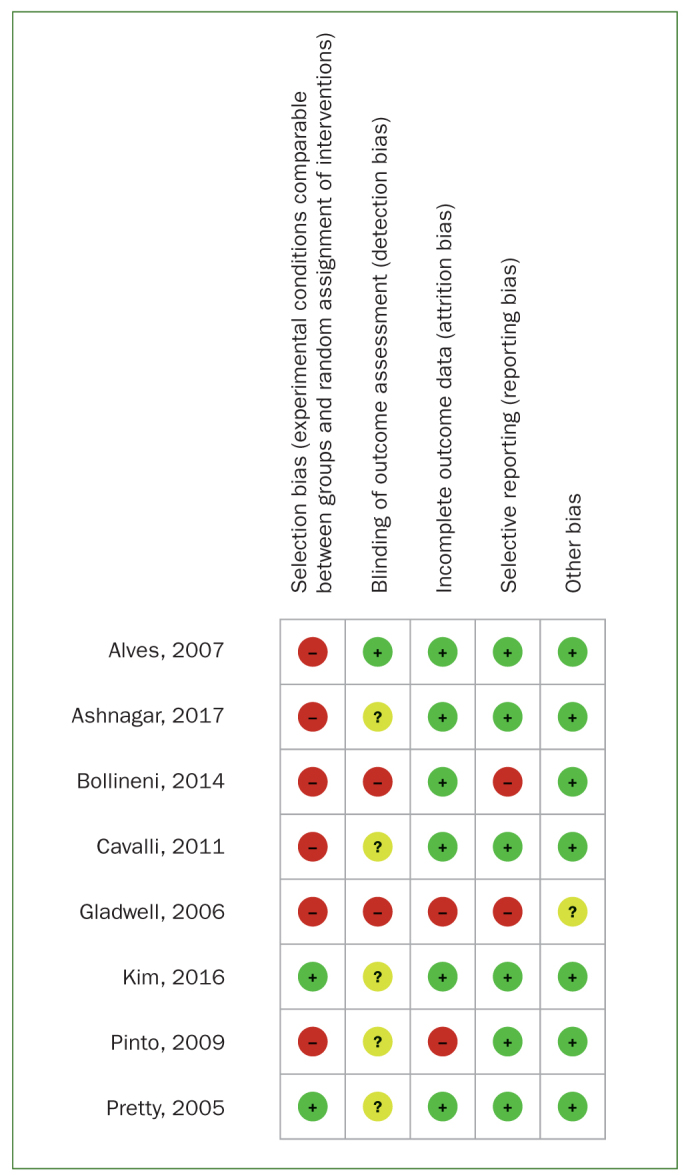
Risk of bias summary for the in vitro studies.

Quality assessment of the included studies is shown in Figs 2 and 3 and Tables 2a and 2b. The RCT^24^ was judged to be at overall high risk of bias. Six of the in vitro studies were classified as having a high risk of bias^2,3,8,10,16,35^ and two as having a moderate risk.^23,38^ Common reasons for downgrading were lack of randomisation procedures, blinding, and attrition bias.

**Table 2a:** Quality assessment of in vitro studies

First author, year	Experimental conditions comparable between groups and random assignment of interventions (selection bias)	Blinding of assessors performing the tests (performance bias)	Losses or non-inclusion of specimens (attrition bias)	Selective reporting of results (reporting bias)	Other bias	Overall risk
Ashnagar, 2017	High	Unclear	Low	Low	Low	High
Kim, 2016	Low	Unclear	Low	Low	Low	Unclear
Bollineni, 2014	High	High	Low	High	Low	High
Cavalli, 2011	High	Unclear	Low	Low	Low	High
Pinto, 2009	High	Unclear	High	Low	Low	High
Alves, 2007	High	Low	Low	Low	Low	High
Gladwell, 2006	High	High	High	High	Unclear	High
Pretty, 2005	Low	Unclear	Low	Low	Low	Unclear

**Table 2b:** Risk of bias assessement of the in vivo study

First author, year	Sequence generation (selection bias)	Allocation concealment (selection bias)	Blinding of participants and personnel (performance bias)	Blinding of outcome assessors (detection bias)	Incomplete outcome data (attrition bias)	Selective reporting (reporting bias)	Other sources of bias	Overall risk
Knösel, 2007	Unclear	Unclear	High	High	Low	High	Unclear	High

### Descriptive Results

#### Clinical trial

The only clinical trial that met the inclusion criteria evaluated the effect of combined home and in-office bleaching on colour changes in 19 participants with visible inactive WSL after orthodontic treatment.^24^ Colour changes were registered by a colorimeter and patient satisfaction was evaluated using a questionnaire. The results, although this trial was graded as having a high risk of bias, showed that the therapeutic scheme could camouflage the WSL and that the patients were pleased with the aesthetic outcome of bleaching.

#### Laboratory studies

The remaining studies were performed in vitro and several concentrations, products and techniques for bleaching were applied as detailed in Table 1a. Among the outcome measures, visual examination, enamel microhardness and degree of demineralisation were the most common. The effect on colour change was addressed in three studies,^8,16,23^ two with high and one with moderate risk of bias, whose results indicated that bleaching reduced colour disparities between sound and carious enamel and that addition of remineralising agents did not affect the whitening properties. Studies on enamel microhardness and histology showed that the mineral loss caused by bleaching could be reduced by the presence of fluorides,^8,16^ calcium^10^ and casein phosphopeptide–amorphous calcium phosphate (CPP-ACP).^23^ Five studies addressed the question of whether bleaching can make teeth more vulnerable for caries development.^2,3,10,35,38^ Four studies at high risk of bias and one study at moderate risk reached the overall conclusion that neither home- nor in-office bleaching of permanent teeth was associated with an increased susceptibility to caries/enamel demineralisation or erosion. In two studies having high risk of bias,^2,3^ laser-activated bleaching did not seem to affect enamel microhardness or caries risk when compared to conventional bleaching with hydrogen or carbamide peroxide.

#### Qualitative synthesis of the included studies

There was considerable methodological heterogeneity across the studies with large differences in interventions, participants (type of teeth) and endpoints. Thus, a meta-analysis was not feasible for any combination of the included studies. Likewise, a funnel plot to disclose potential publication bias was not possible due to the limited number of included studies.

## Discussion

Tooth bleaching has gained interest in recent decades as a key component of aesthetic dentistry. The current technologies are based on home-bleaching, either with products containing carbamide peroxide (CP) or hydrogen peroxide (HP) at concentrations between 3% and 10%, or in-office bleaching, with concentrations ranging from 15% to 38%. However, recent systematic reviews have not detected any major differences in whitening efficacy between the at-home and in-office strategies,^11,12^ although tray-delivered CP gels seem to perform slightly better than HP-based products.^28^ Tooth sensitivity and gingival pain are commonly reported side-effects of tooth bleaching procedures in adults.^18,27^ Thus, it is suggested that bleaching should be restricted to patients with good oral hygiene and be followed by fluoride application in order to enhance renewed mineral uptake.^4^

To the best of our knowledge, this is the first systematic review examining the effect of bleaching as a method for the management of post-orthodontic white spot lesions. Previous reviews on tooth bleaching implemented a more general approach,^15,20,45^ and focused on study design^5^ or adverse effects.^43^ Our findings were inconclusive and compromised by the limited number of studies that met the inclusion criteria, as well as the comparatively low quality of research. It is noteworthy that of the 9 papers evaluated, only one was an RCT. Furthermore, in vitro studies cannot always adequately reflect actual clinical conditions. Factors such as oral hygiene habits, diurnal alterations in saliva flow, and dietary habits are examples of parameters that are impossible to adequately simulate in the laboratory. Human saliva has been found to be less associated with enamel demineralisation than artificial saliva,^5^ which was supported by our observation that the studies using human saliva found post-bleaching measurements of enamel microhardness similar to those at baseline. Thus, a waiting period with natural remineralisation from saliva and self-applied fluoride toothpaste over a period of at least 3 to 6 months after debonding is advocated before any additional treatment options for WSL should be considered.^36^ It should also be noted that the American Academy of Pediatric Dentistry (AAPD) guidelines^37^ discourage the use of full-arch cosmetic bleaching for patients in the mixed dentition.

One clinical and two laboratory studies addressed the primary research question. The results showed that bleaching of WSL can diminish the colour disparities between carious and non-affected areas, but the certainty of the evidence was very low. Moreover, low-quality in vitro data indicated that the presence of fluoride or any other remineralising agent did not impair the whitening effect. The second research question could not be answered in the present review, as only in vitro studies were available. A recent meta-analysis has, however, shown that no significant changes in enamel microhardness appeared when using a 10% carbamide peroxide bleaching gel over a 21-day period.^45^ Although the laboratory data assessed in our study reconfirmed the fact that bleaching did not increase the risk of further demineralisation or decrease enamel microhardness, no firm conclusions can be drawn from studies with a high risk of bias. Therefore, the main conclusion of the present systematic review is that the efficacy of bleaching as a method to manage post-orthodontic white spot lesions lies in a knowledge gap. Similar conclusions were also reached by Höchli et al,^20^ who investigated the therapeutic and adverse effects of different interventions to treat post-orthodontic white spot lesions. According to the authors, although fluoride varnish seemed to be effective, the need for further research was pointed out.

Consequently, the need for future randomised controlled trials involving post-debonding lesions using objective endpoints such as white spot scores, light fluorescence, or impedance spectroscopy, is emphasised. Patients’ subjective perceptions should be also investigated via questionnaires, since the literature has shown that bleaching treatment produces positive changes in young participants’ oral health quality of life in terms of smiling, laughing, and showing teeth without embarrassment.^25^

The effects of adding fluoride and calcium to the bleaching agents were also inconclusive. The combination of carbamide peroxidase and remineralising products resulted in reduced mineral loss when applied to sound enamel,^8,10,16^ but this was not verified when artificial caries lesions were treated.^35^ The problem of extrapolating laboratory findings to clinical settings showed that adverse effects of carbamide peroxide on enamel evident in specimens bleached in vitro were not seen in situ, and that the presence of saliva could prevent the demineralising effect of bleaching gel in situ.^22^ Moreover, the inherently limited ability of in vitro studies to evaluate long-term results is beyond doubt; in turn, this makes the exploration of the time factor unfeasible. The rationale behind using lasers to assist bleaching is that a heat/light source may enhance the peroxide action and maximise the benefit of bleaching without requiring several lengthy sessions for the patient. With the limited information available, we found no support for adding lasers to in-office bleaching. Two systematic reviews have recently confirmed no significant differences in tooth colour change or tooth sensitivity when in-office bleaching gels with and without light were compared.^29,42^

As in most systematic reviews, the considerable variations in study protocols (application time, number of bleaching sessions, product concentration) and reported endpoints indicated a high degree of clinical and methodological heterogeneity, preventing a meta-analysis and making conclusions difficult. Thus, for future studies, employing standardised methodology to evaluate bleaching products would be beneficial. Nevertheless, the most striking challenge was incomplete reporting and the high risk of bias in the existing literature. In particular, a limited number of materials and undefined origin of teeth in combination with selection and performance bias frequently diminished the quality of the included studies. As shown in Tables 1a and 1b, incomplete reporting of data was also a frequent problem.

A limitation of this review is the inclusion of in vitro studies and only one clinical study. This rendered robust clinical conclusions impossible. The decision for including in vitro studies was also based on presenting all published investigations to clinicians and future researchers, irrespective of study design.

## Conclusion

The findings from the present systematic review could neither support nor refute bleaching as an effective method for the management of post-orthodontic white spot lesions. The need for further prospective in vivo studies is support by the fact that most of the studies in this field are in vitro and that there is little solid scientific evidence of low risk of bias.
